# Effects of Walnut Bark Extract on the Human Platelet Aggregation, Adhesion, and Plasmatic Coagulation In Vitro

**DOI:** 10.1155/2023/5644803

**Published:** 2023-01-03

**Authors:** Asmae Amirou, El Mahdi Razzok, Abdelkhaleq Legssyer, Abderrahim Ziyyat, Mohammed Aziz, Mohamed Bnouham, Younes Zaid, Mohamed Berrabah, Hassane Mekhfi

**Affiliations:** ^1^Laboratory of Bio-Resources, Biotechnology, Ethnopharmacology and Health, Mohammed the First University Faculty of Sciences, Oujda, Morocco; ^2^Laboratory of Epidemiology, Clinical Research, and Public Health, Mohammed the First University, Faculty of Medicine and Pharmacy, Oujda, Morocco; ^3^Research Center of Abulcasis University of Health Sciences, Rabat, Morocco; ^4^Department of Biology, Faculty of Sciences, Mohammed V University, Rabat, Morocco; ^5^Laboratory of Mineral Solid and Analytical Chemistry, Mohammed the First University Faculty of Sciences, Oujda, Morocco

## Abstract

Thrombosis is the formation of a clot within a blood vessel. Antithrombotic drugs are used for treating thrombosis, which can be the cause of hemorrhage. Currently, there is a need to discover novel antithrombotic drugs. Walnut is widely used to treat a wide range of health complaints. In this study, walnut bark extract was tested in hemostasis parameters: platelets adhesion, aggregation, and plasmatic coagulation in human blood. The crude aqueous extract of walnut bark was prepared by infusion and tested *in vitro* on hemostasis. Through blood collection from healthy volunteer donors, we studied different parameters of the primary hemostasis: platelet adhesion on the collagen-coated surface under flow, ADP, collagen, thrombin, and arachidonic acid-induced platelet aggregation, and of the secondary hemostasis by measuring prothrombin time (PT) and activated partial thromboplastin (APTT) parameters. All experiments are realized in the absence and presence of the extract and repeated at least twice. The obtained data showed that the extract (1 and 2 mg/mL) significantly (*p* < 0.001) reduced the activated platelet adhesion on the collagen-coated surface. In the same way, the effect of the extract on platelet aggregation seems to depend on its concentration and on the nature of the agonist. The strongest inhibition of aggregation was observed in the case of collagen at 1 mg/mL, while there was no observed effect on arachidonic acid-induced aggregation. Moreover, the extract (1 mg/mL) affects the extrinsic, intrinsic, and common pathways of the human blood coagulation cascade by extending significantly (*p* < 0.001), both PT and APTT times. This study provides evidence that walnut bark extract, by its antiadhesive, antiaggregant, and anticoagulant activities, could be considered as a serious source of biological compounds for the prevention and treatment of thrombosis.

## 1. Introduction

Hemostasis is a physiological vital process but might become a physio-pathological one. Normally, in case of vascular injury, it consists to stop bleeding by the formation of a blood clot. It is divided into three steps: primary hemostasis involving local vasoconstriction, platelet adhesion, and platelet aggregation. Secondary hemostasis allows the formation of fibrin and the achievement of the development of a red blood clot. The last step, fibrinolysis, is the process of proteolytic digestion of fibrin aimed at dissolving the thrombus and restoring the blood flow. Unfortunately, under some physio-pathological conditions, the same process of hemostasis may be triggered causing abnormal thrombosis formation and leading to cardiovascular complications [1, 2]. Many antithrombotic treatments exist which are both effective but also have many side effects. Nowadays, there is an increased interest in using natural products derived from plants or animals for the development of new compounds (antiplatelet, anticoagulant, and antithrombotic drugs) with less bleeding and toxic side effects [3]. Currently, medicinal plants are gaining much interest in pharmaceutical industries and served as a part of the primary medical in the treatment of the thrombotic complications. Several studies have reported antiplatelet, anticoagulant [4–6], and antithrombotic effects on many plants [3, 7–10].

In medicinal flora, Morocco is one of the most important diversities of the Mediterranean basin, particularly for tree species including olive, fig, and walnut [11]. The most important areas of walnut were identified in the Rif, High, and Medium Atlas mountains and in arid regions in Southeastern Morocco [12]. The consumption of walnut is increasing due to the high concentration of natural antioxidants that have been reported as being protective against cancer [13] and may also decrease the risk of cardiovascular diseases [14]. Several pharmacological studies have shown the beneficial effect of the consumption of walnut such as antioxidant, antidiabetic, anti-inflammatory, antihypertensive, and anticancer [15–17]. Many studies [18–20] stated that walnut is an excellent source of phenolic compounds such as phenolic acids, namely gallic, ellagic, syringic, caffeic, p-coumaric, and tannins. Other researchers determined its phenolic profile: quercetin 3-O-galactoside (the major compound), quercetin 3-O-pentoside derivative, and other derivatives of quercetin [21].

In a previous report, we have already demonstrated the antiaggregant and anticoagulant effect of walnut root bark extract on blood rat *in vitro* and *ex vivo* [22]. Moreover, in our recent study, flavonoid-rich fractions of walnut have exhibited a significant preventive antithrombotic effect on an experimental model of thrombosis in mice [23]. To our knowledge, there is no similar study conducted on hemostasis in humans, the goal of this study is to investigate the impact of walnut bark extract on the human hemostasis especially on *in vitro* platelet adhesion and aggregation, and on plasmatic coagulation.

## 2. Methods

### 2.1. Plant Collection and Preparation

The root bark of walnut (*Juglans regia* L, Juglandaceae) was collected in southern Morocco. The plant was identified by Professor Mohammed FENNANE, an expert botanist, from the Scientific National Institute, Rabat (Morocco), and recorded under a voucher number: HUMPO 149. The bark of the walnut was washed with water and dried. The dried bark was powdered using a blender and kept in a dark until the time of use.

The crude aqueous extract of walnut bark (WBE) was prepared by infusion. The infusate was then filtered and evaporated by a rotary evaporator (Heidolph Instruments, Germany) at 45°C. The yield of extraction was 11.8%.

### 2.2. HPLC Analysis of Walnut Bark Extract

HPLC analyses were performed using Waters Alliance 2695 system (Waters Co., Milford, MA, USA) with an analytical C18 column (250 mm × 4.6 mm, particle size 5 *μ*m). Walnut bark extract (20 *μ*L) was separated at the room temperature and a flow rate of 1 mL/min via two phases: the mobile phase A composed of 1% of formic acid and Milli-Q water [10 : 90 (v/v)] and the mobile phase B composed of Milli-Q water, acetonitrile and formic acid [40 : 50 : 10 (v/v/v)]. Samples were eluted with the following gradient: 0 min: 88% A + 12% B, 20 min: 70% A + 30% B, 30 min: 0% A + 100% B, and 45 min: 88% A + 12% B. The standard references used are rutin, kaempferol, quercetin, catechol, and gallic acid. Chromatograms were acquired at two wavelengths (283 and 365 nm) according to the absorption maxima of analyzed compounds. Each compound was identified by its retention time.

### 2.3. Isolation of Human Platelets for Aggregation Assay

Whole blood was obtained by venipuncture from healthy volunteer donors who accepted and signed the consent form. The study was approved by the Scientific Ethic Committee for the Biomedical Research of the Medicine and Pharmacy Faculty of Oujda, Morocco (Protocol No 04/2021).

Whole blood was anticoagulated with acid-citrate-dextrose (v/v ratio 9 : 1) (citric acid 130 mM, trisodium citrate 170 mM, dextrose 4%), centrifuged for 10 min at 170 g to obtain platelet-rich plasma (PRP). The PRP was centrifuged for 5 min at 80 g to eliminate residual blood cells and for 15 min at 1500 g to obtain platelets. In the end, platelets were suspended in Tyrode buffer (NaCl 134 mM, KCl 2.9 mM, MgCl_2_ 1 mM, NaHCO_3_ 12 mM, Na_2_HPO_4_ 0.34 mM, Hepes 10 mM, glucose 5 mM, bovine serum albumin 0.3%, pH 7.4) to adjust their amount to 350000 cells/mm^3^ [24]. During experiences, final washed platelets (WP) were kept at room temperature and used on the same day.

### 2.4. Platelet Aggregation Assay

The platelet effect of WBE was evaluated *in vitro* by using an aggregometer system (Helena Biosciences Europe, Beaumont, Texas, USA) measuring light transmission amount through the WP suspension. Under a constant temperature (37°C) and stirring (1000 rpm), the WP was preincubated for 2 min with different concentrations of WBE (0.25, 0.5, 1 mg/mL). Platelet aggregation was induced by adding adenosine 5′-diphosphate (ADP, 5 *μ*M), collagen (5 *μ*g/mL), thrombin (0.5 U/mL), and arachidonic acid (20 *μ*M). The signal was recorded for at least 10 min, and results were expressed as a percentage of maximal platelet aggregation.

### 2.5. Rhodamine-Based Assay for Human Platelet Adhesion

Individuals' healthy donors were recruited with informed consent at the Cheikh Zaïd Hospital in Rabat, Morocco. This study was approved by the Local Ethics Committee of Cheikh Zaid Hospital, Rabat, Morocco (Project: CEFCZ/PR/2020-PR04), and complies with the Declaration of Helsinki. The relationship between the activation of platelets and the pretreatment for 5 min with the extract of 1 and 2 mg/mL of WBE was assessed in an *ex vivo* perfusion system of human whole blood exposed to collagen-coated surfaces under flow. For all conditions (except negative control), platelets were prestimulated with 0.5 U/mL of thrombin. The ratio of activated platelets to total platelets (activated and non-activated platelets) was examined by double immunofluorescence using rhodamine-conjugated P-selectin antibody (activated platelets) and fluorescein isothiocyanate-conjugated platelet membrane glycoprotein antibody (GPIIb/IIIa, total platelets). In brief, glass capillaries were coated overnight at 4°C with 250 *μ*g/mL of fibrillar equine type 1 collagen (Chronolog). Two milliliters of sodium citrate anticoagulant were mixed with 200 *μ*L of cell suspension (500 × 10^3^) followed by incubation with rhodamine 6G for 15 min at 37°C [25].

Stained samples were then perfused simultaneously in glass capillaries at a shear rate of 300/second for 5 min. After a washing step, images of the surfaces with adherent platelets were captured using a digital camera connected to a Nikon Eclipse NI-Motorized Microscope System (Plan 10 × 0.25, Melville, NY, USA). Platelet adhesion was quantified by morphometric analysis (NIH, Bethesda, MD), and platelet adhesion data were expressed as the percentage of capillary surface covered by platelets.

### 2.6. Human Plasmatic Coagulation Assay

Blood was collected directly into tubes containing anticoagulant (sodium citrate 3.8) from healthy volunteers. Blood was centrifuged for 10 min at 1500 g to obtain platelet-poor plasma (PPP). The prothrombin time (PT) and activated partial thromboplastin time (APTT) measurements were carried out using a semiautomatic coagulometer (Thrombostat, Behnk Elektronik, Norderstedt, Germany).

First, an aliquot of WBE was incubated with PPP (50 *μ*L) for 5 min at 37°C. For the PT test, 100 *μ*L of PT reagent (Neoplastine) was added to 50 *μ*L of WBE-PPP mixture, and clotting time was then measured. For the APTT test, 50 *μ*L of reagent (C.K. Prest) was added to 50 *μ*L of WBE-PPP mixture and incubated for 1 min. To start the measurement of APTT, 50 *μ*L of calcium chloride (0.025 M) was added. Heparin (0.4 U/mL) was used as a positive control for the coagulation test [22].

### 2.7. Statistical Analysis

All the results were expressed as mean ± standard deviation (SD), and statistical analysis was performed by using GraphPad Software (version 5.01, GraphPad Software, Inc.). *p* values of less than 0.05 (*p* < 0.05) were considered as significant.

### 2.8. Reagents

Adenosine 5′-diphosphate (ADP) purchased from Verum Diagnostica GmbH (Munich, Germany), collagen from collagen calf skin type III, Sigma (USA), thrombin from Sigma (Germany), and arachidonic acid from CALBIOCHEM (USA). APTT reagent C.K. Prest® from Diagnostica Stago, France, PT reagent: Neoplastine® Cl provided by Diagnostica Stago, France, Rhodamine 6G (Sigma-Aldrich), fibrillar equine type 1 collagen (Chronolog).

## 3. Results

### 3.1. HPLC Profiles of Walnut Bark Extract

**Figure 1:**
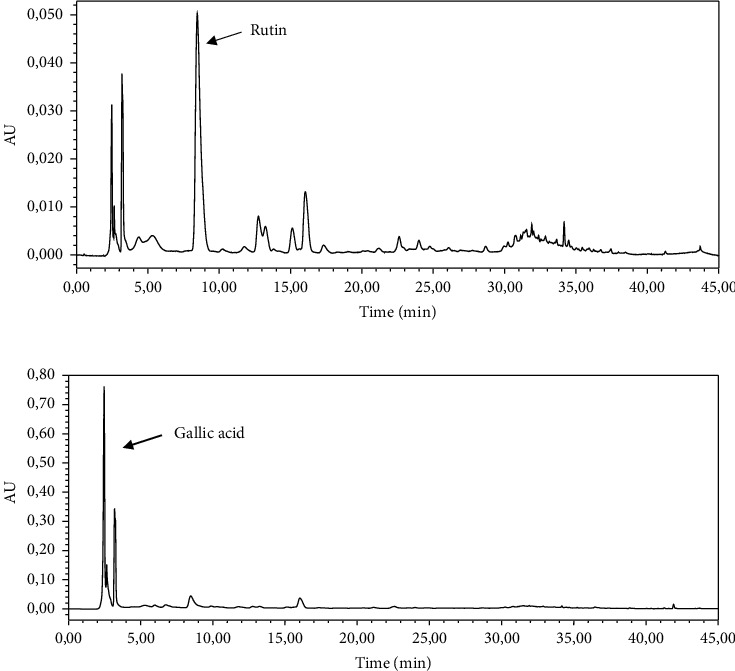
HPLC profiles of walnut bark extract obtained at 365 nm (a) and 283 nm (b).

HPLC with C18 columns is the most used technique for the analysis of polyphenols in different parts of the plant. In order to identify the compounds, present in the walnut bark crude extract, five standard molecules, with different retention times, were used as follows: gallic acid (2.78 min), catechol (6.49 min), rutin (8.31 min), quercetin (23.32 min), and kaempferol (31.45 min). The HPLC profile obtained has revealed several peaks with two major ones. Compared to standard retention times, these two peaks are identified as rutin and gallic acid (Figure 1). However, the others remain unidentified.

### 3.2. Walnut Bark Extract Effect on Platelet Aggregation

WBE (0.25, 0.5, and 1 mg/mL) was evaluated *in vitro* on a human platelet aggregation triggered by different agonists: ADP (5 *μ*M), collagen (5 *μ*g/mL), thrombin (0.5 U/mL), and arachidonic acid (20 *μ*M).

**Figure 2:**
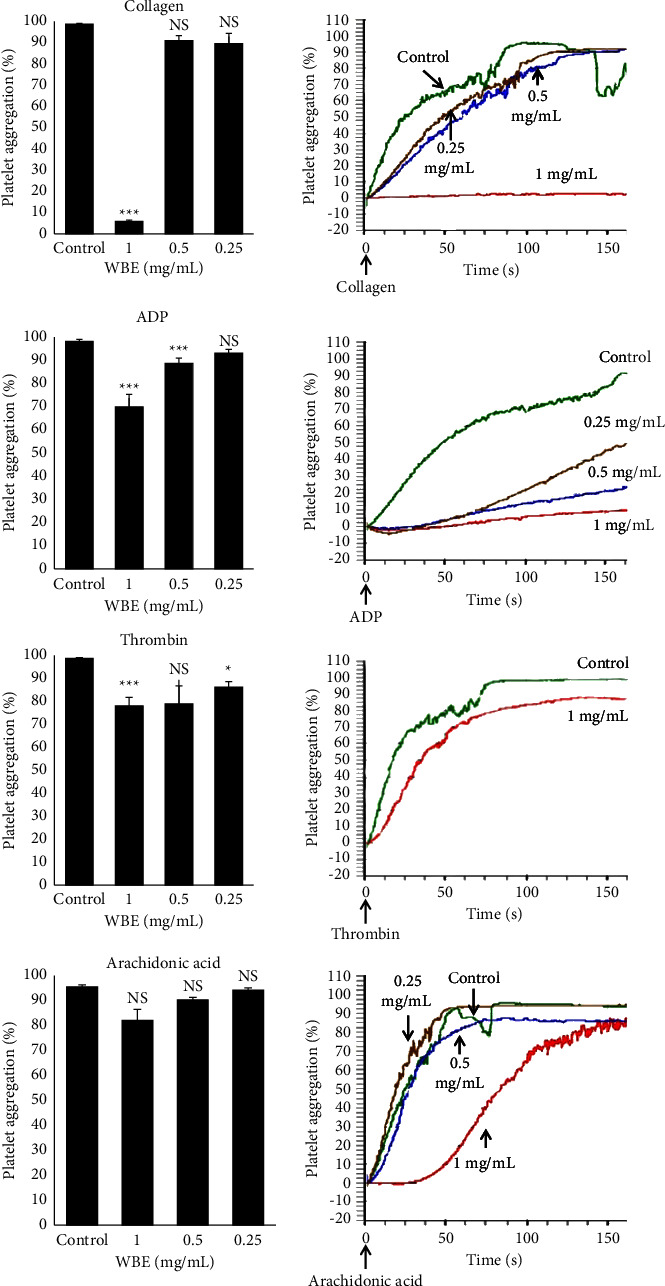
*In vitro* platelet aggregation traces and histograms showing the effect of 0.25, 0.5, and 1 mg/mL of walnut bark extract (WBE) on collagen (5 *μ*g/mL), ADP (5 *μ*M), thrombin (0.5 U/mL), and arachidonic acid (20 *μ*M) induced platelet aggregation. ^*∗*^*p* < 0.05, ^*∗∗∗*^*p* < 0.001, ns: nonsignificant when compared to control samples (washed platelets incubated without extract) *n* = 3−6.

As shown in Figure 2, the effect of the extract seems depending on its concentrations and/or the used agonist. Indeed, 1 mg/mL of the WBE suppressed significantly (*p* < 0.001) aggregation in case of thrombin (20.95% of inhibition) and ADP (28.85% of inhibition) and especially in collagen-induced aggregation (more than 90% of inhibition). In contrast, platelets stimulated with arachidonic acid showed no significant difference (*p* > 0.05).

### 3.3. Walnut Bark Extract Inhibited Human Platelet Adhesion

**Figure 3:**
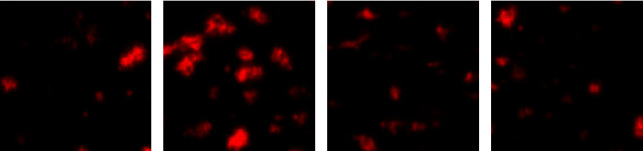
Platelet adhesion on collagen was evaluated under flow conditions after 5 min. (a) Negative control, (b) positive control, and (c) and (d) walnut bark extract (WBE) at 1 and 2 mg/mL, respectively, *n* = 3.

Platelet adhesion, which is the first step in the hemostasis process, plays a determinant role in thrombus formation. Human whole blood was exposed *in vitro* to collagen-coated surfaces under flow, and the platelet adhesion was quantified by calculating the surface covered (platelets amount adhered to collagen, %). In nonactivated platelet (negative control), the platelet adhesion was only 14 ± 2.64% compared to 42 ± 4.35% in thrombin-activated platelet (positive control) (Figures 3(a) and 3(b), respectively). The pretreatment of blood samples with the WBE, at 1 and 2 mg/mL, significantly reduced the surface covered to 22 ± 3.6% (*p* < 0.001) and 16.33 ± 0.57% (*p* < 0.001), respectively (Figures 3(c) and 3(d)), compared to the positive control.

### 3.4. Walnut Bark Extracts Prolonged PT and APTT

The blood clotting activity of WBE was investigated by determining PT and APTT to evaluate globally the extrinsic and intrinsic pathways, respectively.

Table 1 shows that WBE at 1 mg/mL significantly extended (*p* < 0.001) PT (22.54 ± 2.66 s) and APTT (91.42 ± 14.05 s).

As expected, heparin (0.4 U/mL) used as a reference anticoagulant drug, significantly (*p* < 0.001) prolonged both blood clotting times.

## 4. Discussion

Walnut (*Juglans regia* L, Juglandaceae family) is the widest-spread tree nut in the world. It is commonly known as Persian, English, or common walnut. In Morocco, it is well known as “Aljawaz” or “Gargae” for the fruit and “Swak” for the bark and root bark. Walnut is a rich source of nutraceutical and pharmaceutical properties that exhibit numerous health benefits and pharmacological effects [16].

Under normal physiological conditions, platelets circulate close to the endothelium without forming any stable adhesion contact, due to its antiadhesive property. However, in some physio-pathological situations, they become abnormally hyperactive [26] and lead to the formation of clots within blood vessels, contributing to the onset of thrombosis and increasing the rate of cardiovascular and cerebrovascular complications [27]. In the case of a vascular injury, the endothelium is damaged, and platelets are immediately exposed to the subendothelial collagen, which is a major thrombogenic component of this layer. Then, platelets form stable adhesion contacts with this collagen and other adhesives macromolecules [28]. In the present study, we have evaluated the *in vitro* effect of walnut bark extract on human platelet adhesion and aggregation, and on plasmatic coagulation.

To investigate *in vitro* the effect of WBE on the adhesion process, we performed a flow-based adhesion assay over a collagen-coated surface. Our results have shown that the pretreatment of the blood with WBE significantly reduced the number of platelets adhered to the collagen surface. These data mean that some constituents present in this extract interact between collagen and its platelet receptors glycoprotein (GP) VI and integrins *α*2*β*1, so preventing the platelet adhesion to the subendothelial matrix. This outcome is in accordance with a recent study by Kao and Kung [29] that showed that the major compound of *Juglans regia*: juglone reduced thrombus formation on collagen-coated surfaces under arterial shear rates. Our result is interesting because it concerns the initial step of the hemostasis process, leading to the thrombus formation. Indeed, platelet adhesion is followed immediately by the platelet activation, granules secretion, aggregation, and plasmatic coagulation. In this context, we examined *in vitro* the WBE extract on platelet aggregation. For this study, washed platelets were prepared from healthy volunteers without any antiaggregant or anticoagulant medications. They were exposed to the WBE, and then, platelet aggregation was triggered by adding various agonists such as ADP, thrombin, collagen, and arachidonic acid. Our results revealed that WBE, at 1 mg/mL, markedly suppressed platelet aggregation induced by collagen, ADP, and thrombin, but not arachidonic acid. Rywaniak et al. [30] previously examined the walnut impact on aggregation and demonstrated that the preincubation of whole blood platelets with *Juglans regia* husks extract significantly decreased platelet aggregation induced by ADP and had no significant effect on either collagen or arachidonic acid stimulated aggregation. However, according to these same authors, platelet-rich plasma incubation with *Juglans regia* was found to have no significant effect on platelet aggregation induced by ADP. Furthermore, Meshkini and Tahmasbi [31] showed that walnut hull extract displayed a distinct inhibitory effect on platelet aggregation in a dose-dependent manner induced by thrombin. This discrepancy in results may be linked to the part of the plant used for extraction, the type of tested extract, and certainly to the blood sample (whole blood, platelet-rich plasma, or washed platelets).

This result lets us to suggest that WBE may interact negatively with one or more platelet receptors. Indeed, platelets become active and aggregate with each other after the attachment of their agonists on specific glycoprotein receptors and throughout different intracellular signaling pathways. Some of these hypotheses are well described in the literature. The integrin *α*2 *β*1 and GPVI are the major collagen platelet receptor: the integrin *α*2 *β*1, with a major role in platelet adhesion, and the GPVI, principally responsible for signaling and platelet activation [32]. P2Y1 and P2Y12 are ADP receptors coupled to G protein and involve the binding of fibrinogen to glycoprotein GP IIb/IIIa receptors on platelets. Activation of P2Y1 leads to the activation of phospholipase C, inducing intracellular calcium mobilization, granule release of other mediators, and platelet shape change whereas the activation of P2Y12 couples with Gi reducing adenylyl cyclase activity and producing aggregation response [8]. Regarding thrombin, it is well known that human platelets express protease-activated receptors 1 (PAR1) and 4 (PAR4) as thrombin receptors [33]. As a result, walnut bark does not have a specific suppressive effect on human platelets, but it can be attributed to the activation of platelet GP IIb/IIIa, which is the common last step of platelet aggregation. The role of the GP IIb/IIIa receptor in the thrombotic process is mainly associated with the subunit IIIa that affects the ability to bind to the fibrinogen molecule [34]. Indeed, a recent study by Kao et al. [29] confirms this idea and reported that juglone (5-hydroxyl-1,4-naphthoquinone), a phenolic compound produced by walnut trees, inhibited platelet aggregation and GP IIb/IIIa activation caused by various agonists: thrombin, collagen, and U46619 (thromboxane A2 receptor agonist).

In parallel with the primary hemostasis, the plasmatic coagulation constitutes a second part in the completion of the thrombus generation. Here, we explored the impact of WBE on PT and APTT of the human platelet-poor plasma. These two clot times are the main indicators of the extrinsic, intrinsic, and common pathways of the coagulation cascade. PT assesses the extrinsic pathway factors (FVII) and common pathway factors (FX, FV, and FII as well as fibrinogen) activities. APTT is determined to evaluate the intrinsic pathway factors (FXII, FXI, FIX, and FVIII) and common pathway factors (FX, FV, FII, and fibrinogen) [35]. At 1 mg/mL, WBE shows an anticoagulant effect by enhancing both PT and APTT. This result means that the extract could reduce the activity of one or several coagulation factors implicated in intrinsic and extrinsic pathways whereas the prolongation of the common pathway duration indicates probably an inhibition of thrombin-mediated fibrin formation. This hypothesis agrees with our previous study conducted on rat's blood [22], where WBE significantly decreased the plasmatic fibrinogen level *in vitro* and *ex vivo* studies.

Unlike purified fractions, the crude aqueous extract is a complex mixture of bioactive compounds that may act separately or in synergy. Numerous researches reported the presence of various phytochemicals in different parts of walnut. In our study, the HPLC analysis of walnut bark extract revealed the presence of rutin and gallic acid. The extract contains also coumarins, isocoumarins anthocyanins, tannins, ascorbic acid, paracomaric acid, and essential fatty acids [18]. In addition, a wide range of phenolic acids and flavonoids was mentioned such as myricetin, gallic acid, caffeic acid, quercetin, rutin, quercetin galactoside, and quercetin derivatives, and a marker compound: juglone (5-hydroxy naphthoquinone) was described in the bark of walnut [16, 18, 36]. Consequently, the antiplatelet and anticoagulant activities of walnut bark might be attributed to their components especially flavonoids, phenolic acid, and coumarins. According to several studies, polyphenols exert many health effects, the most important being the antioxidant, anti-inflammatory, antiplatelet, anticoagulant, antithrombotic, and anti-ischemic effects [37, 38]. To elucidate the mechanism of action by which these compounds act on the hemostasis process, many suggestions are proposed such as blocking the formation of thromboxane, increasing the intraplatelet concentration of cAMP and cGMP, and inhibiting the cleavage of phosphatidylinositol and inhibition of phospholipase C [39]. Certain flavonoids such as quercetin, rutin, and catechin influenced the level of nitric oxide (a natural antiplatelet molecule) and blocked the glycoprotein IIb/IIIa complex, thus blocking the platelet activation and aggregation [40, 41]. For the anticoagulant effect of WBE, the mechanism of action may be related to the inhibition of thrombin activity, which generates normally fibrinogen from fibrin. Bijak et al. [42] have demonstrated that the polyphenolic grape seeds extract inhibited the proteolytic activity of thrombin, observed as inhibition of thrombin-induced fibrinogen polymerization.

Our results show, for the first time, the multipotential effect of WBE on the adhesion, aggregation platelet, and coagulation system and clearly suggest that this plant could be considered as a promising nutraceutical in the prevention of cardiovascular thrombotic events. Further investigations are needed to support this hypothesis like exploring the antithrombotic and fibrinolytic activities of WBE.

## 5. Conclusion

In summary, our study has revealed that WBE significantly inhibited *in vitro* human platelet adhesion, aggregation, and prolonged cloth times: PT and APTT. These results may be attributed in part to rutin and gallic acid, and compounds present in the extract and support the hypothesis that WBE may exert a beneficial effect on human health by preventing some thrombotic complications. Further studies are needed to confirm the mechanism of action of WBE in thrombotic activity by using other experimental models of thrombosis.

## Figures and Tables

**Table 1:** Effect of walnut bark (*Juglans regia*) on human PT and APTT.

	Concentration	PT (s)	APTT (s)
Control	(1 : 1/v,v)	13.44 ± 1.54	38.22 ± 6.1
WBE (mg/mL)	1	22.54 ± 2.66^*∗∗∗*^	91.42 ± 14.05^*∗∗∗*^
Heparin (U/mL)	0.4	21.52 ± 2.77^*∗∗∗*^	>300

Mean ± SD, PT: prothrombin time, APTT: activated partial thromboplastin time, ^*∗∗∗*^*p* < 0.001. >300: no coagulation. *n* = 5.

## Data Availability

The data used to support the study are included in the paper.
